# Relationship between Brain-Derived Neurotrophic Factor and Schneiderian First Rank Symptoms in Antipsychotic-Naïve Schizophrenia

**DOI:** 10.3389/fpsyt.2013.00064

**Published:** 2013-07-02

**Authors:** Sunil Vasu Kalmady, Ganesan Venkatasubramanian, Venkataram Shivakumar, Dania Jose, Vasanthapuram Ravi, Bangalore N. Gangadhar

**Affiliations:** ^1^Department of Psychiatry, The Schizophrenia Clinic, National Institute of Mental Health and Neuro Sciences, Bangalore, India; ^2^Translational Psychiatry Laboratory, Cognitive Neurobiology Division, Neurobiology Research Centre, National Institute of Mental Health and Neuro Sciences, Bangalore, India; ^3^Department of Neurovirology, National Institute of Mental Health and Neuro Sciences, Bangalore, India

**Keywords:** schizophrenia, antipsychotic-naïve, BDNF, Schneiderian first rank symptoms

## Abstract

Neurodevelopmental aberrations influenced by neurotrophic factors are among the important paradigms to understand schizophrenia pathogenesis. Among various neurotrophic factors, Brain-Derived Neurotrophic Factor (BDNF) is strongly implicated by previous research studies. Evaluating co-morbidity free, antipsychotic-naïve schizophrenia patients for BDNF levels and examining the correlates of this factor with symptoms might facilitate elucidation of its pathogenetic role without confounds of potential influencing factors. In this study, 59 co-morbidity free, antipsychotic-naïve schizophrenia patients were compared with 60 healthy controls for serum BDNF levels. In addition, the relationship between Schneiderian First Rank Symptoms (FRS) and BDNF level in patients was examined. As a group, schizophrenia patients (28.8 ± 11.7 ng/mL) had significantly lower serum BDNF than healthy controls (34.9 ± 8.2 ng/mL) after controlling for the potential confounding effects of age and sex (*F* = 7.8; *p* = 0.006). Further analyses revealed FRS status to have significant effect on plasma BDNF after controlling for the potential confounding effects of age and sex (*F* = 4.5; *p* = 0.01). Follow-up *post hoc* analyses revealed FRS(+) patients to have significant deficit in plasma BDNF level in comparison with healthy controls (*p* = 0.002); however, FRS(−) patients did not differ from healthy controls (*p* = 0.38). Our study observations add further support to the role for BDNF in schizophrenia pathogenesis and suggest a potential novel link between deficient BDNF and FRS.

## Introduction

Neurodevelopmental model postulates schizophrenia as a behavioral outcome of an aberration in brain development processes that begins long before the onset of clinical symptoms and is caused by a combination of genetic and environmental factors (1). Gene-environment interaction especially involving genetic factors and obstetric complications have been put forth as one of the important mechanisms that increase the risk toward schizophrenia (2). Specifically, the genetic factors that interact with obstetric complications have been postulated to encompass hypoxia-ischemia regulated genes and Brain-Derived Neurotrophic Factor (BDNF) gene is one among them (2). Given the increasing emphasis on the role of neurotrophic factors in the neurodevelopmental pathogenesis of schizophrenia (3), BDNF has attracted significant attention in schizophrenia research studies.

The compelling role for BDNF in the pathogenesis of schizophrenia is supported by various lines of evidence [see for reviews (4, 5)]. BDNF, the most widely distributed neurotrophin in the central nervous system, is highly expressed in brain regions that are critically implicated in the pathogenesis of schizophrenia – the prefrontal cortex and hippocampus (6). BDNF has been demonstrated to interact with various neurotransmitter systems that are implicated in schizophrenia, such as dopamine, glutamate, serotonin, and GABA (7).

Post-mortem studies have reported decreased levels of BDNF protein in the hippocampus (8) and prefrontal cortex (9) in brains of schizophrenia patients. Furthermore, significantly decreased cerebrospinal fluid (CSF) levels of BDNF have been documented in antipsychotic-naïve, first episode psychosis patients (10). Interestingly, Pillai et al. (10) also demonstrated a significant positive correlation between the CSF and plasma BDNF protein levels supporting the view that measurement of blood/plasma BDNF levels reflect the brain tissue levels (11).

Peripheral level of BDNF in schizophrenia patients has been evaluated in many studies (12). Though an earlier meta-analytic evaluation of these study findings supported decreased peripheral levels of BDNF in schizophrenia patients (12), further studies on antipsychotic-naïve patients have been recommended by a recent review (5) to avoid the potential confounding effects of antipsychotic treatment. Apart from medication status, symptom profile of schizophrenia patients is another important factor with which BDNF level has been linked. Significant negative correlation has been reported between BDNF level and severity of positive symptoms in few studies (10, 13). In relation to this observation, it is important to note that met-BDNF allele [the genotype that is shown to be associated with reduced BDNF level (14)] has been shown to be associated decreased volume (15), lower metabolic ratios of *N*-acetyl aspartate and glutamate (16) of the hippocampus, and as well as with reduced volume of the right inferior parietal lobule (17) – the brain regions that are implicated in the genesis of positive symptoms in schizophrenia (18). Interestingly, the right inferior parietal lobule abnormalities has been repeatedly shown to be associated with Schneiderian First Rank Symptoms (FRS) in many studies (19–22). However, it has to be acknowledged that the anatomical and pathophysiological basis of positive symptoms and Schneiderian FRS are still very poorly understood, and several brain regions other than those mentioned above have been proposed to contribute to them. Since BDNF has been reported to influence brain development until adolescent/early adult period, it is plausible BDNF deficit might result in reduced volumes/aberrations in these specific brain regions.

In this study, we evaluated serum BDNF in antipsychotic-naïve schizophrenia patients (*N* = 59) in comparison with healthy controls (*N* = 60). As reviewed above, very few studies that have evaluated the symptom correlates of BDNF in schizophrenia suggest significant negative correlation between BDNF and positive symptoms (10, 13) as well as link between BDNF and certain brain regions that are shown to underlie the genesis of FRS (17). To ascertain this relationship further, in this study, we have evaluated antipsychotic-naïve schizophrenia patients in comparison with healthy controls for serum BDNF; also, we concurrently assessed the Schneiderian FRS in patients. We hypothesized that schizophrenia patients with FRS will show deficient BDNF level in comparison with healthy controls while those without FRS will not differ significantly.

## Materials and Methods

Patients attending the clinical services of the National Institute of Mental Health and Neurosciences (India), who fulfilled DSM-IV criteria for schizophrenia and were never treated with any psychotropic medications including antipsychotics and not having substance abuse [*n* = 59; age = 31 males], were examined in this study. The diagnosis of schizophrenia was established using Mini International Neuropsychiatric Interview Plus (23), which was confirmed by another psychiatrist through an independent clinical interview. The details related to illness onset and antipsychotic-naïve status as well as substance use was carefully ascertained by reliable information obtained from at least one reliable adult relative (first-degree). Psychotic symptoms were assessed using Scale for Assessment of Positive Symptoms (SAPS) and Scale for Assessment of Negative Symptoms (SANS).

First Rank Symptoms were examined using a comprehensive semi-structured interview (22) as per the established descriptions (24); these 11 symptoms described by Mellor included the following: audible thoughts, voices arguing, voices commenting, thought insertion, thought withdrawal, thought broadcast, made feelings, made impulses, made volitions, somatic passivity, and delusional percept (24). In addition, the presence of FRS was independently ascertained by a qualified psychiatrist by a comprehensive mental status examination. Depending upon the presence of at least one FRS, schizophrenia patients were classified into those with FRS [FRS(+); *N* = 36] and those without FRS [FRS(−); *N* = 23].

Healthy controls (*n* = 60) (age = 26.4 ± 4.7 years; 29 males), who volunteered for study, were screened to rule out any psychiatric diagnosis using the MINI as well as a comprehensive mental status examination. None of the controls had family history of psychiatric disorder in first-degree relatives.

Patients and controls did not have features suggestive of alcohol abuse/dependence. History related to substance use in patients was carefully ascertained by reliable information obtained from at least one reliable adult relative (first-degree). None used stimulant or opiate drug. None had history or clinical feature suggestive of neurological/medical disorder. None had abnormal movements as assessed by Abnormal Involuntary Movements Scale. Physical activity status of all study subjects was quantified using the Simplified Indian Diabetes Risk Score. Clinical assessments and blood sample collection were performed on the same day before starting antipsychotics. After complete description of study to the subjects, written informed consent was obtained. The Institute’s ethics committee approved the study.

Blood samples were collected from all subjects between 08:00 and 09:00 h (a.m.) after 12-h overnight fast. Blood was drawn from an ante-cubital vein into vacutainer tubes (Becton and Dickinson, USA). Using a serum separator tube (SST™ II Advance, BD Vacutainer^®^, NJ, USA) samples were collected, mixed well by inversion, and allowed to clot for 30 min before centrifugation for 15 min at 1000 × *g*. Serum was separated, aliquoted, and stored at −80° C. Quantitative sandwich enzyme immunoassay of serum BDNF was done using commercially available ELISA kit with sensitivity<20 pg/mL (R&D Systems, MN, USA). All samples were coded and analyzed by the same investigator, who was blind to the clinical situation. Serum was examined instead of plasma in order to avoid irreproducible results due to BDNF contained in platelets and released by platelet activation. Briefly, samples (diluted 20-fold) and standards (concentration 62.5–4000 pg/mL) are incubated in 96-well microplate pre-coated with BDNF specific monoclonal antibody followed by incubation with enzyme-linked monoclonal antibody specific for BDNF. After three washes, the reaction was developed with tetramethylbenzidine (TMB) and stopped with sulfuric acid. The absorbances were measured with an automated microplate reader at 450 nm (corrected for optical imperfections at 620 nm) [The standard curves of the ELISA assays and the subject-wise BDNF values are provided in the Data Sheet S1 and S2 in Supplementary Material respectively].

Data analysis was performed using the SPSS-11.0 using the following statistics after ascertaining the normality of the data distribution: Student’s *t*-test (two-tailed), chi-square test, analysis of variance (ANOVA), and analysis of covariance (ANCOVA).

## Results

**Figure 1:**
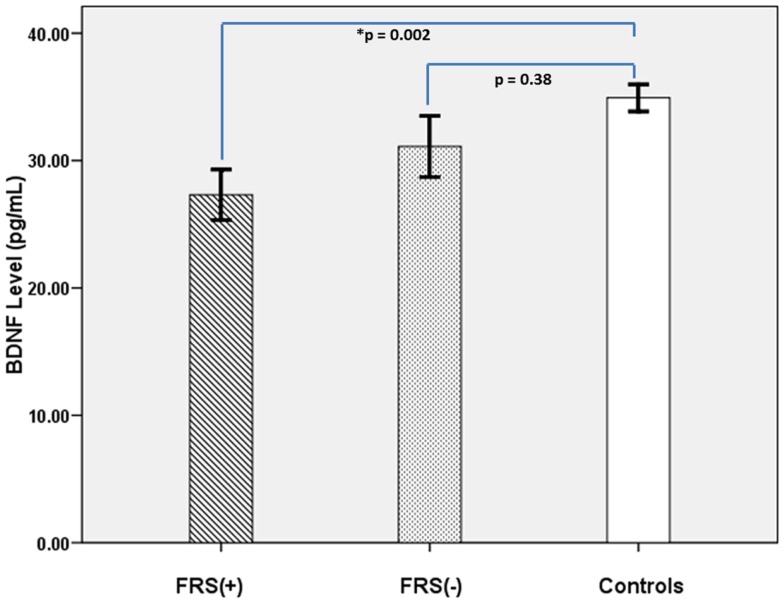
**It shows comparative profile of serum BDNF levels in FRS(+) patients (*N* = 36), FRS(−) (*N* = 23), and healthy controls (*N* = 60)**.

The characteristics of the study subjects are given in Table 1. FRS(+) patients, FRS(−) patients and healthy controls, as a group, did not differ significantly in sex ratio and physical activity scores (*p* > 0.3). Both FRS(+) and FRS(−) patients were significantly older than healthy controls; however, between them, FRS(+) and FRS(−) patients did not significantly differ in age. As a group, schizophrenia patients (28.8 ± 11.7 ng/mL) had significantly lower serum BDNF than healthy controls (34.9 ± 8.2 ng/mL) after controlling for the potential confounding effects of age and sex (*F* = 7.8; *df*  = 117; *p* = 0.006). Further analyses revealed FRS status to have significant effect on plasma BDNF after controlling for the potential confounding effects of age and sex (*F* = 4.5; *df*  = 6, 112; *p* = 0.01) (Table 1; Figure 1). Follow-up *post hoc* analyses revealed FRS(+) patients to have significant deficit in plasma BDNF level in comparison with healthy controls (*p* = 0.002); however, FRS(−) patients did not differ from healthy controls (*p* = 0.38). There was no significant effect of sex of the subject on plasma BDNF in this analysis. SAPS (*r* = 0.01; *p* = 0.93) and SANS (*r* = 0.06; *p* = 0.69) total scores did not show any significant correlation with serum BDNF level.

**Table 1:** **Comparative profile of schizophrenia patients with and without FRS and healthy controls**.

Characteristics	FRS(+)	FRS(−)	Controls	Statistic	*p*
*N*	36	23	60		
Age (years)*	31.7 ± 6.8	30.4 ± 7.5	26.4 ± 4.7	*F* = 12.2	<0.001
Sex ratio (M:F)^$^	17:19	14:09	29:31	χ2 = 1.3	0.53
Age at onset of psychosis (years)^#^	28.1 ± 7.6	29.4 ± 7.1	–	*t* = 0.6	0.52
Duration of untreated psychosis (months)^#^	28.1 ± 27.0	29.2 ± 41.3	–	*t* = 0.1	0.52
Physical activity score*	24.2 ± 6.5	25.7 ± 7.3	23.0 ± 9.1	*F* = 0.9	0.39
BDNF (ng/mL)**	27.3 ± 11.8	31.1 ± 11.5	34.9 ± 8.2	*F* = 4.5	0.01

*ANOVA; ^$^chi-square test; ^#^independent samples t-test; **ANCOVA controlling for potential confounding effects of age and sex.

## Discussion

Our study finding of significantly deficient serum BDNF level in antipsychotic-naïve schizophrenia patients replicates previous similar reports (13, 25–27). The deficient BDNF level seems to be predominantly contributed by schizophrenia patients with FRS; to the best of our knowledge, this is the first time report of relationship between FRS and deficient BDNF in schizophrenia. Some of the previous studies have demonstrated significant negative correlation between BDNF level and severity of positive symptoms (10, 13). In our study, we did not find any significant correlation total positive symptom score; however, in comparison with healthy controls, patient with FRS showed significantly deficient BDNF level whereas those without FRS did not differ significantly. The FRS assessed in this study encompassed audible thoughts, voices arguing, voices commenting, thought insertion, thought withdrawal, thought broadcast, made feelings, made impulses, made volitions, somatic passivity, and delusional percept – these symptoms are unified by the striking breach in one’s ability ability to differentiate “self” from “non-self.” It is possible that the component of delusions and hallucinations as emphasized by the FRS might be linked with the BDNF and this might explain the heterogeneity in previous reports. Moreover, in this context, it has to be acknowledged that a correlation between BDNF and FRS does not imply causality. The possibility that third factors may be involved should be recognized, while identifying these factors may not be possible.

Imaging studies examining the neural correlates of FRS in schizophrenia patients have demonstrated relationship with abnormalities involving parietal cortex (19, 22), parahippocampal gyrus, and posterior cingulate cortex (28). It is interesting to note that these brain regions have been shown to be affected by BDNF. For example, healthy 66Met allele carriers had significantly deficient inferior parietal lobule (17) and parahippocampal gyrus volumes (29). In a large study examining 258 adult twins and their non-twin siblings, it was demonstrated that BDNF polymorphism significantly contributed to the variation in white matter integrity in the posterior cingulate gyrus by accounting for around 90–95% of total variance in fractional anisotropy (30).

It is noteworthy that 66Met allele has been associated with schizophrenia risk [see for reviews (4, 5)]. Moreover, 66Met allele is associated with decreased levels of BDNF (31). Thus, it is possible that deficient levels of BDNF might have led to aberrations involving critical brain regions (like inferior parietal lobule, parahippocampal, and posterior cingulate gyri) resulting in genesis of FRS in schizophrenia. Our study observations offer preliminary support to this hypothesis.

Examination of antipsychotic-naïve schizophrenia patients, ensuring the uniform timing of collection of blood sample matches with some of the methodological strengths of the previous similar studies. In addition, we matched the patients and controls for physical activity status which could be another potential confounding factor. Significant difference in age between patients and controls can be construed as a potential limitation, since increasing age has been associated with decreased BDNF level. However, the significance of BDNF difference persisted even after controlling for age; moreover, we did not observe any significant correlation between age and BDNF level in this study. Measuring BDNF level in serum than CSF might be considered as another limitation especially since we attempt to infer the peripheral BDNF levels as indicative of brain levels; nonetheless, it is important to note that previous reports have supported this assumption (10, 32).

In summary, our study observations add further support to the role for BDNF in schizophrenia pathogenesis. These findings also support the possibility that the BDNF changes in early life that perhaps render individuals more susceptible to schizophrenia also continue into adulthood with adverse impact on neurodevelopmental trajectory. In addition, a potential novel link between deficient BDNF and Schneiderian FRS is suggested by these findings. This needs to be evaluated further with concurrent analyses involving genotype as well as peripheral level of BDNF along with brain imaging studies in the same cohort of schizophrenia patients.

## Authors Contribution

Authors Ganesan Venkatasubramanian, Vasanthapuram Ravi, and Bangalore N. Gangadhar conceptualized and designed the study. Authors Ganesan Venkatasubramanian, Sunil Vasu Kalmady, and Venkataram Shivakumar collected the data. Sunil Vasu Kalmady, Dania Jose, and Venkataram Shivakumar performed the analyses. Authors Ganesan Venkatasubramanian and Sunil Vasu Kalmady managed the literature search and wrote the first draft of manuscript. Authors Ganesan Venkatasubramanian, Vasanthapuram Ravi, and Bangalore N. Gangadhar revised and optimized further versions of the manuscript. All the authors have contributed to and have approved the final manuscript.

## Conflict of Interest Statement

The authors declare that the research was conducted in the absence of any commercial or financial relationships that could be construed as a potential conflict of interest.

## Supplementary Material

The Supplementary Material for this article can be found online at http://www.frontiersin.org/Schizophrenia/10.3389/fpsyt.2013.00064/abstract
